# The Potential of Generative AI to Support Medicare Decision Making

**DOI:** 10.1093/geroni/igaf122.1638

**Published:** 2025-12-31

**Authors:** Neil Charness, Walter Boot, Sara Czaja, Wendy Rogers, Joseph Sharit, Emily Langston, Xin Yao Lin

**Affiliations:** Weill Cornell Medicine, New York, New York, United States; Weill Cornell Medicine, New York, New York, United States; Weill Cornell Medicine, New York, New York, United States; University of Illinois Urbana-Champaign, Champaign, Illinois, United States; University of Miami, Coral Gables, Florida, United States; Florida State University, Tallahassee, Florida, United States; Weill Cornell Medicine, New York, New York, United States

## Abstract

In 2022, ∼ 99% of American adults age 65+ yr were enrolled in Medicare, a complex, difficult to use insurance system. We describe a multi-pronged approach to assessing the potential value of AI to support Medicare decision-making processes that includes querying subject matter experts who provide Medicare advice, assessing Medicare users’ knowledge, preferences, and abilities through interviews and while interacting with knowledge sources, and evaluating existing AI tools for accuracy, reliability, and conciseness. In this presentation we present findings about the accuracy and reliability of digital assistants in answering Medicare questions, a new tool for assessing Medicare knowledge, and a study to assess preferences and performance with Medicare information sources. Generative AI (ChatGPT, Bard) were highly accurate (>90%) and reliable as well as superior to the average Medicare beneficiary, and much superior to digital home assistants (Alexa and Google Home Assistant). The Medicare Proficiency Questionnaire proved to be a short, reliable, and valid scale of older adults’ Medicare knowledge, differentiating between Medicare enrollees and non-enrollees. Prior access to the Medicare website and the Medicare and You Handbook mediated the relationship between knowledge and education level as well as knowledge and Medicare enrollment status, suggesting that understanding Medicare information sources may be critical for designing AI decision support tools and training. We also describe early results from an observational study with Medicare-enrolled and unenrolled older adults examining preferences for, and performance with, the Medicare.gov website, the Medicare and You Handbook, and Gemini (a Large Language Model).

